# Variation in dementia screening outcomes: the influence of primary care providers’ occupations and knowledge, attitudes, skills

**DOI:** 10.1186/s12875-025-02886-y

**Published:** 2025-05-28

**Authors:** Yanglin Qiu, Xinyu Fan, Yanjuan Wu, Shibin Wang, Wenyan Tan, Jing Liao

**Affiliations:** 1 Department of Medical Statistics, School of Public Health, Sun Yat-sen University, Guangzhou, China; 2 Sun Yat-Sen Global Health Institute, Institute of State Governance, Sun Yat-sen University, Guangzhou, China; 3 Guangdong Mental Health Center, Guangdong Provincial People’s Hospital (Guangdong Academy of Medical Sciences), Southern Medical University, Guangzhou, China

**Keywords:** Dementia screening, Knowledge, Attitudes, Skills, Primary care providers

## Abstract

**Background:**

Dementia is a growing concern in China. Primary care screening is proposed as a timely and cost-effective way to identify potential cases, while its implementation relies on primary care providers’ (PCPs’) knowledge, attitudes, and skills. Our study aimed to investigate whether dementia screening outcomes vary according to the PCPs’ occupations and how PCPs’ knowledge, attitudes, and skills are associated with screening outcomes.

**Methods:**

A two-stage dementia screening of residents aged 65 years and over in Guangdong, China was conducted using multistage cluster sampling. 252 PCPs, who completed questionnaires, were included in the analysis, along with 2823 older adults screened by them. Screening outcomes included the positive rate of screening, and positive predictive value (PPV). PCPs’ knowledge was assessed by the Dementia Knowledge Assessment Scale, attitudes by the Dementia Care Attitudes Scale, and skills by a validated self-designed questionnaire. Multilevel logistic regression was used to assess the associations of PCPs’ occupations, knowledge, attitudes, and skills with dementia screening outcomes.

**Results:**

Knowledge scores in dementia care differed significantly (*P* = 0.005) among PCPs of different occupations, while attitudes and skills did not. The positive screening rate had no significant association with PCPs’ occupations. Public health physicians (OR = 2.927, 95%CI: 1.091–7.854) and nurses (OR = 3.712, 95%CI: 1.141–12.069) had a higher PPV than general practitioners (GPs). Higher dementia-care skills score (OR = 1.024, 95%CI: 1.004–1.046) was associated with a higher positive rate of screening. Positive attitudes towards dementia care was associated with a lower positive rate of screening (OR = 0.948, 95% CI: 0.905–0.994) and a higher PPV (OR = 1.114, 95% CI: 1.007–1.234).

**Conclusions:**

In community settings, involving different occupations of PCPs besides GPs in dementia screening and systematically promoting dementia-care skills training and positive attitudes towards dementia care are important to improve the effectiveness of dementia screening and management.

**Supplementary Information:**

The online version contains supplementary material available at 10.1186/s12875-025-02886-y.

## Introduction

Dementia is a growing public health concern worldwide, with China bearing the highest dementia burden reaching 15.07 million in 2020 [1]. As a significant contributor to disability, the impact of dementia extends beyond the individual to encompass their family members. Early detection of dementia, as the basis for all treatment efforts, holds the promise of reducing the risk of dementia and its consequences [2]. However, under-detection or low screening rate of dementia, which means that a proportion of people with mild cognitive impairment or suspected dementia may not be screened, remains a widespread problem, particularly in developing countries such as China [3]. It is estimated that 85.8% of people aged 60 and over were not screened for dementia in China [4], the high under-detection rate requiring effective means to address this issue.

Screening in primary care centers has been proposed as a timely and cost-effective approach to the early identification of older adults with dementia, particularly in resource-constrained settings with limited access to specialised dementia care, such as memory clinics or general hospitals. The accessibility gap can be well filled by primary care providers (PCPs) in community-based primary care centers, who can perform basic dementia screening tasks and refer those in need to high-tier specialists [1, 5]. Screening for dementia mainly consists of two stages [6]. In the first stage, the dementia screening test conducted by PCPs is used to identify cognitively impaired older adults at high risk of dementia. These screened positive will then undergo the second stage of diagnosis, which is conducted by psychiatrists based on clinical guidelines (Chinese guideline for the diagnosis and treatment of Alzheimer’s disease dementia) [7].

A wide variation is evident in the detection rate of cognitive impairment in the first stage of screening in China, with a range of 2.5–36.6% [8–12]. Even in a city like Shenzhen, there is a wide variation in the positive rates reported by community-based screening programs (i.e. 2.5% and 21.9%) [8, 12]. Besides factors associated with screened older adults, characteristics of PCPs who conducted these screening may also have an impact on screening outcomes. Previous studies indicate that the majority of Chinese PCPs have limited knowledge and training about dementia, thus that often hold negative attitudes towards screening and lack of relevant skills to identify high risk patient in time [13]. The Capabilities, Opportunities, Motivations, Behavior (COM-B) model assumes that each individual’s behavior is influenced by capability (psychological or physical), opportunity (social or physical), and motivation (reflective or automatic) [14]. The model serves as a theoretical framework for understanding and promoting behavioral change, with applications spanning individual health promotion, policy development and assessment, and identification of barriers and facilitators to behavioral change. Our qualitative study, guided by COM-B model, further indicates that PCPs’ poor capability due to inadequate knowledge and skills about dementia, limited capability due to insufficient knowledge and skills regarding dementia, reduced opportunity due to unclear referral and management pathways, and low motivation stemming from negative attitudes are primary barriers to effective community-based dementia screening [15]. However, to our best knowledge, no study has directly quantified the association between PCPs’ knowledge, attitudes, and skills of dementia and dementia detection practices.

The evidence is unclear regarding whether the dementia screening detection rate differs across various PCPs. Dementia screening is primarily conducted by general practitioners (GPs), who are often the first point of contact for patients with chronic conditions such as hypertension and diabetes and oversee annual health check-ups for adults aged 65 and older [15]. Increasingly, however, other PCPs, including community nurses, public health physicians, and social workers, are participating in dementia screening in China [16]. Compared to GPs, these providers typically have fewer clinical responsibilities and often maintain closer relationships with patients and their families. Nevertheless, the knowledge and skills of these providers regarding dementia—particularly among social workers who lack formal medical training [17]—may lower than those of GPs.

Therefore, our study aimed to investigate whether dementia screening outcomes vary according to the PCPs’ occupations and how PCPs’ knowledge, attitudes, and skills are associated with screening outcomes. Quantifying these associations would capture essential elements to provide evidence to optimise dementia screening practices and improve their accuracy in primary care.

## Methods

### Study design and setting

This study is a cross-sectional questionnaire-based survey conducted after a two-stage dementia screening organised by the Guangdong Mental Health Center in Guangdong from October to December 2021. Guangdong Province, ranking as the top four regions in China for the prevalence of Alzheimer’s disease and related dementias [18], was selected as a pilot site to promote dementia screening in primary care centers as part of the Healthy China Action initiative (2019–2030).

The two-stage screening includes: In the first stage, all older adults were screened using the Community Screening Interview for Dementia (CSI-D) [19] and Ascertain Dementia 8 (AD8) [20] by PCPs at local primary healthcare centers or township hospitals/village clinics in each selected city. Those with a CSI-D score ≤ 24 or an AD8 score ≥ 2 were considered positive and invited to undergo the second stage of dementia diagnosis, conducted by qualified psychiatrists following the Chinese guideline for the diagnosis and treatment of Alzheimer’s disease dementia [7] to determine whether the older adults actually have dementia.

After completing the first stage of screening, PCPs were invited to participate an online questionnaire survey. Questionnaires were entered into the REDCap electronic platform by the researchers and rechecked to avoid logical errors. They were required to fill in their basic demographic information and complete questionnaires regarding knowledge, attitudes, and skills in dementia care. All participants provided informed consent. By evaluating PCPs’ knowledge, attitudes and skills in dementia care, we could understand their current status in these aspects and the associations of these aspects with the dementia screening outcomes.

### Study sample

**Fig. 1:**
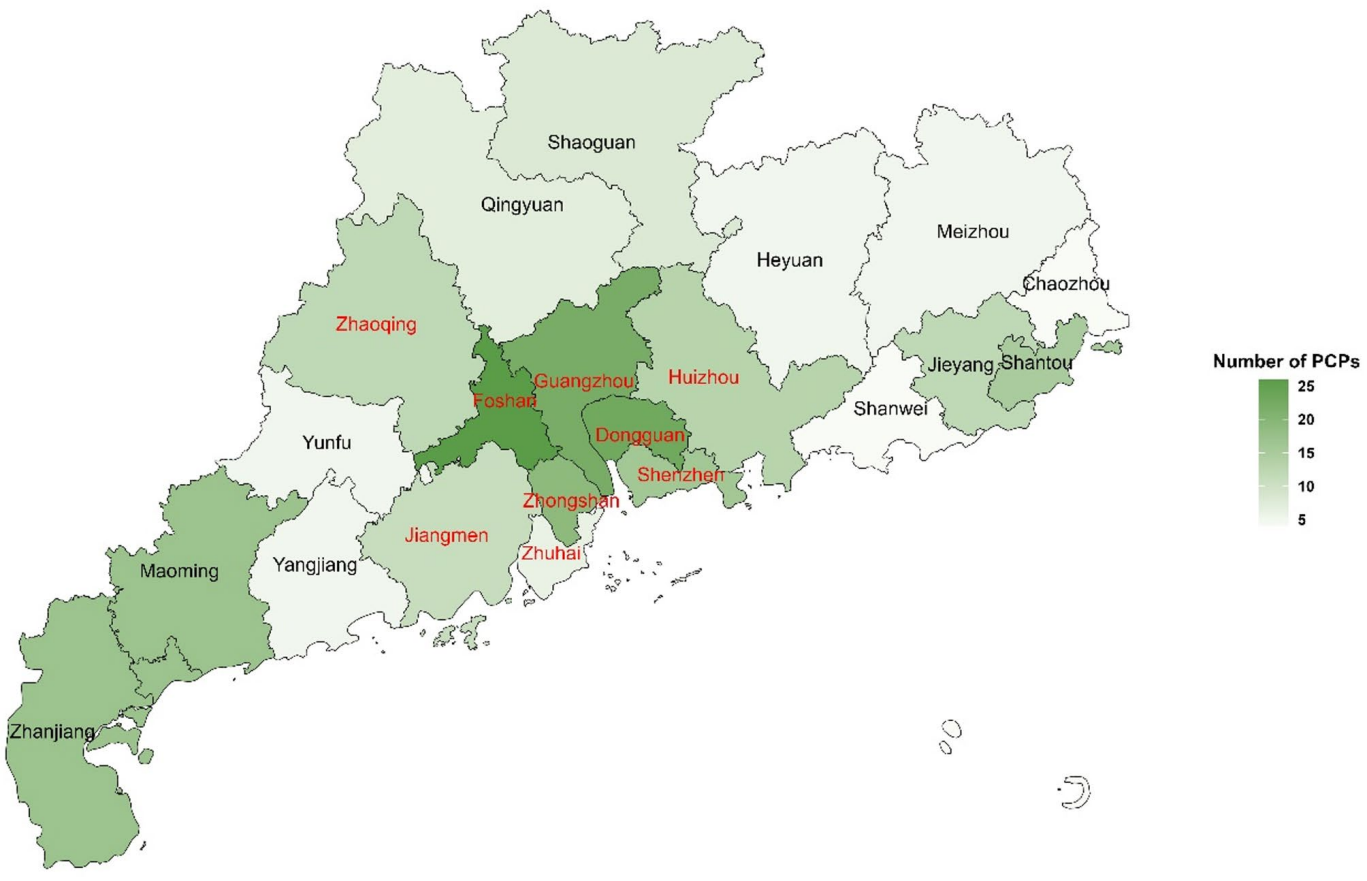
The distribution of primary care providers (PCPs) Note: The PCPs who participated in the survey came from 21 prefecture-level cities in Guangdong Province. The hue of the colors in the graph indicates the number of PCPs, with darker colors representing a higher number of PCPs. Those with labels in red letters are for the Pearl River Delta (PRD) and those with labels in black letters are for the non-Pearl River Delta (NPRD)

The dementia screening was among Chinese older adults aged 65 years and older, who residents in the catchment area of urban primary healthcare centers (*n* = 130) or rural township hospitals/village clinics (*n* = 370) of 72 districts/counties, 21 prefecture-level cities in Guangdong Province. The prefecture-level cities can be divided into the Pearl River Delta (PRD) region and the non-Pearl River Delta (NPRD) region based on their level of economic development (Fig. 1), with PRD being economically developed than NPRD.

**Fig. 2:**
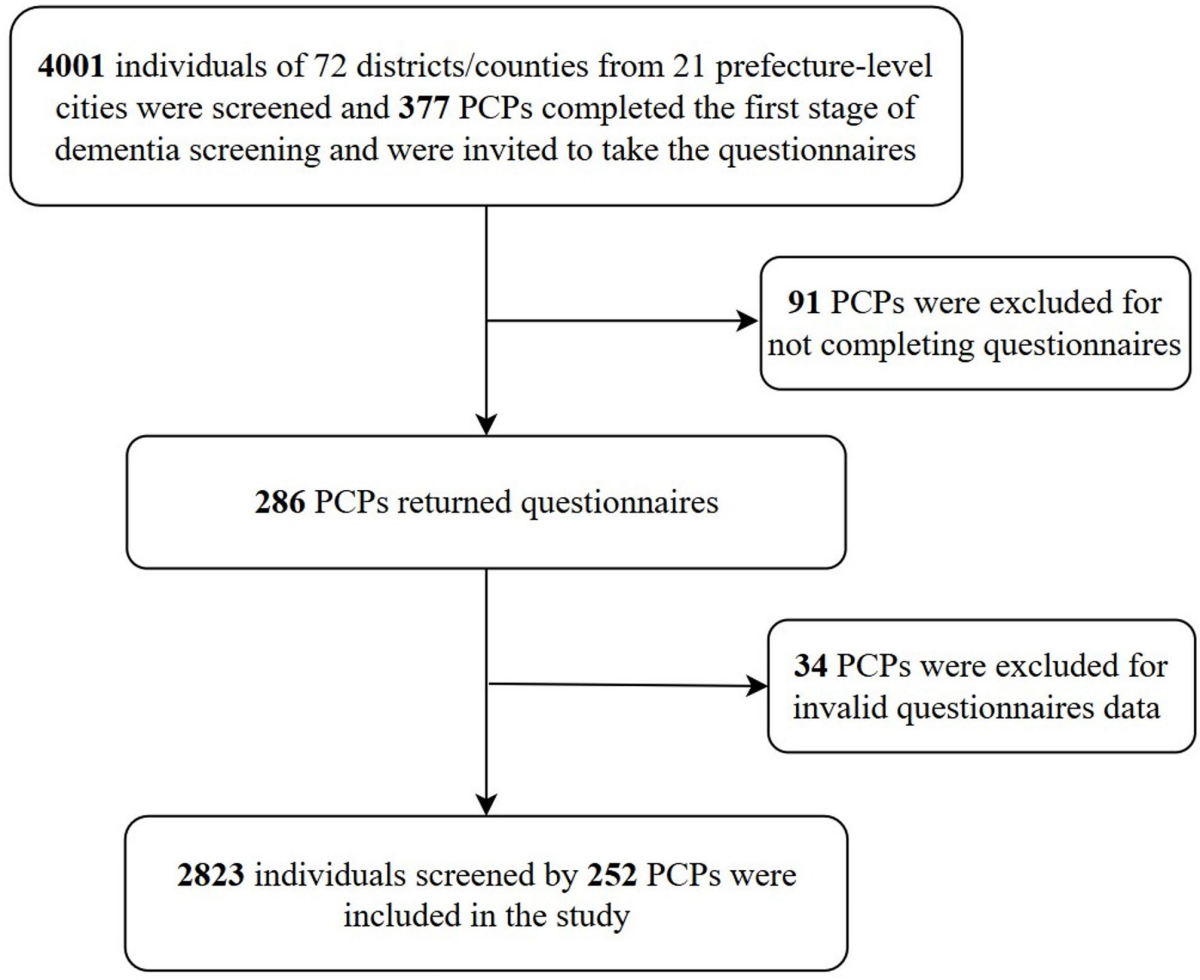
Flowchart for dementia screening by primary care providers (PCPs)

PCPs from all primary healthcare centers or township hospitals/village clinics who participated in the first stage of screening constituted our study sample (*n* = 377). Those who did not complete the questionnaires (*n* = 91), or with invalid data (e.g. questionnaires with consecutive answers to the same option) (*n* = 34) were excluded. A total of 252 PCPs thus was included in the analysis, who screened 2823 older adults. The detailed sample selection flow chart is presented in Fig. 2.

### Measurements of PCPs’ knowledge, attitudes and skills in dementia care

PCPs’ knowledge, attitudes and skills in dementia care were measured respectively by the Dementia Knowledge Assessment Scale (DKAS) [21], the Dementia Care Attitudes Scale (DCAS) [22] and a previously validated self-designed questionnaire by Wang [23].

The DKAS is a tool for measuring dementia-related knowledge, suitable for a range of professionals and students [21]. We used its Chinese version developed by Zhao, which has shown good reliability and acceptable concurrent validity [24]. The scale consists of 25 items, each with 5 response options: false, probably false, probably true, true, and I do not know. The scoring system assigns 2 points for a correct answer of true or false, 1 point for a correct answer of probably true or probably false, and 0 points for an incorrect answer or “I do not know”. The total score ranges from 0 to 50, with higher scores indicating greater knowledge of dementia.

The DCAS is used to assess PCPs’ attitudes towards dementia care, covering different aspects such as early detection, diagnosis, screening, disclosure, guidance, and referral [22]. It consists of 10 items scored on a 5-point Likert scale with responses ranging from 1 (strongly disagree) to 5 (strongly agree). The total score ranges from 10 to 50, with higher scores indicating more positive attitudes towards dementia care.

PCPs’ dementia care skills were measured by assessing their level of confidence in dementia care skills using a 5-point Likert scale developed by Wang [23]. It includes 15 items ranging from 1 to 5 (1 = I can’t do it at all, 2 = I can’t do it well, 3 = I’m not sure, 4 = I can do it probably, 5 = I can do it very well). The total score ranges from 15 to 75, with higher scores indicating greater confidence in dementia care skills.

### Screening outcomes

The primary screening outcomes for our study were: (1) positive rate of screening: proportion of individuals screened positive (i.e., scored ≤ 24 on the CSI-D or ≥ 2 on the AD8) by PCPs among all older adults in the first stage; and (2) positive predictive value (PPV): proportion of individuals further diagnosed as having dementia by qualified psychiatrists in the second stage among all positive screening cases who completed the second stage.

### Covariates

The covariates in this study included characteristics of older adults (age, sex and education level) and characteristics of PCPs. The characteristics of PCPs incorporate both sociodemographic characteristics (age, sex and education level) and occupational characteristics. The occupational characteristics included their occupations (i.e., GP, public health physician, nurse, or social worker), working years (considering the stage of career development of Chinese PCPs and enhancing the interpretability of the results, the working years were grouped into < 3 years, 3–10 years and ≥ 10 years), experience with dementia care (providing dementia diagnosis/treatment/care services in the past year, yes or no), working institution (urban primary care centers or rural township hospitals/village clinics), and location (i.e., PRD or NPRD).

### Statistical analysis

Descriptive statistics, including mean and standard deviation, frequency and percentage were presented for PCPs’ characteristics, knowledge, attitudes, and skills in dementia care, and dementia screening outcomes across occupations. Category variables were used the Chi-square test and continuous variables were used one-way ANOVA to examine differences in characteristics among the four occupational groups. Bonferroni multiple comparison procedures were conducted for subgroup comparisons of knowledge, attitudes and skills scores.

Multilevel logistic regression analysis was conducted to assess the association between PCPs’ occupations and screening outcomes (screened positive in the first stage and PPV in the second stage), with screened older adults modelled at level 1, and corresponding PCPs at level 2. In this study, different PCPs served as random effects and other variables served as fixed effects. For PCPs’ occupations, we chose GPs as the reference given its adequate sample size and it is the main occupation conducting screening. The intra-class correlation coefficient (ICC) for the null model with positive rate of screening as the dependent variable was 28.2%, and the null model with PPV as the dependent variable was 34.7%, suggesting that it is suitable for multilevel logistic regression analysis. We analysed the variables at level 1 (older adults’ characteristics) and the variables at level 2 (PCPs’ characteristics) by placing them stepwise into the model. This approach was employed to adjust for confounding variables and to identify the factors that do affect screening outcomes. Model 1 only included the PCPs’ occupations. Model 2, further included Level-1 covariates associated with screened older adults (age, sex and education level). Model 3 further included other Level-2 covariates related to PCPs’ characteristics (age, sex, education level, working years, experience with dementia care, working institution and location) and their knowledge, attitudes, and skills in dementia care. All analyses were conducted using R version 4.4.0 (R core team, Vienna, Austria), and all tests were two-tailed, with statistical significance set at 0.05.

## Results

### Study sample characteristics

Table 1 showed the characteristics of the PCPs and the screened older adults by PCPs. Most participants in the study were public health physicians (45.6%), followed by GPs (26.6%), nurses (14.7%), and social workers (13.1%). GPs (40.0 ± 6.7 years) were significantly older than the other groups of PCPs. More than half of public health physicians and GPs were male, whereas most nurses and social workers were female. GPs (68.7%) and social workers (60.6%) are undergraduate and above, compared with only 18.9% of nurses. 67.2% of GPs and 59.5% of nurses had 10 or more years of working experience. Only 15.2% of social workers had experience providing dementia diagnosis/treatment/care services, compared with 41.7% of public health physicians and 38.8% of GPs. There was a significant difference in the working institution among different PCPs groups (*P* = 0.014), with the highest proportion of social workers (84.8%) having their workplace in the urban, and the lowest being public health physicians (58.3%). The other details are showed in Table 1.

**Table 1:** Characteristics of PCPs and screened older adults

Characteristics	General practitioner (*n* = 67, 26.6%)	Public health physician (*n* = 115, 45.6%)	Nurse (*n* = 37, 14.7%)	Social worker (*n* = 33, 13.1%)	*P*
**Sociodemographic Characteristics of PCPs**					
Age, mean (SD)	40.0 (6.7)	35.9 (8.2)	36.4 (8.2)	35.6 (7.5)	**0.005**
Sex, n (%)					**< 0.001**
Female	21 (31.3%)	47 (40.9%)	35 (94.6%)	26 (78.8%)	
Male	46 (68.7%)	68 (59.1%)	2 (5.4%)	7 (21.2%)	
Education level, n (%)					**< 0.001**
Below undergraduate	21 (31.3%)	60 (52.2%)	30 (81.1%)	13 (39.4%)	
Undergraduate and above	46 (68.7%)	55 (47.8%)	7 (18.9%)	20 (60.6%)	
Working years, n (%)					**< 0.001**
< 3 years	2 (3.0%)	24 (20.9%)	2 (5.4%)	13 (39.4%)	
3–10 years	20 (29.9%)	44 (38.3%)	13 (35.1%)	8 (24.2%)	
≥ 10 years	45 (67.2%)	47 (40.9%)	22 (59.5%)	12 (36.4%)	
Experienced with dementia care, n (%)					**0.040**
No	41 (61.2%)	67 (58.3%)	25 (67.6%)	28 (84.8%)	
Yes	26 (38.8%)	48 (41.7%)	12 (32.4%)	5 (15.2%)	
Working institution, n (%)					**0.014**
Rural	17 (25.4%)	48 (41.7%)	12 (32.4%)	5 (15.2%)	
Urban	50 (74.6%)	67 (58.3%)	25 (67.6%)	28 (84.8%)	
Location ^1^, n (%)					**0.009**
Non-Pearl River Delta	24 (35.8%)	56 (48.7%)	18 (48.6%)	6 (18.2%)	
Pearl River Delta	43 (64.2%)	59 (51.3%)	19 (51.4%)	27 (81.8%)	
**Characteristics of screened older adults in the first stage (***n* = **2823)**					
Age, mean (SD)	71.3 (5.4)	71.9 (6.0)	72.0 (6.3)	71.2 (5.5)	**0.024**
Sex, n (%)					**< 0.001**
Female	419 (52.3%)	582 (43.7%)	225 (52.6%)	129 (49.4%)	
Male	382 (47.7%)	751 (56.3%)	203 (47.4%)	132 (50.6%)	
Education level, n (%)					**< 0.001**
Primary school and below	482 (60.2%)	743 (55.7%)	228 (53.3%)	109 (41.8%)	
Junior school and above	319 (39.8%)	590 (44.3%)	200 (46.7%)	152 (58.2%)	
**Characteristics of older adults completing the second stage (***n* = **595)**					
Age, mean (SD)	72.7 (5.6)	73.4 (7.3)	74.3 (7.7)	71.9 (6.1)	0.125
Sex, n (%)					0.740
Female	74 (49.0%)	147 (51.2%)	51 (55.4%)	31 (47.7%)	
Male	77 (51.0%)	140 (48.8%)	41 (44.6%)	34 (52.3%)	
Education level, n (%)					**0.029**
Primary school and below	86 (57.0%)	189 (65.9%)	50 (54.3%)	32 (49.2%)	
Junior school and above	65 (43.0%)	98 (34.1%)	42 (45.7%)	33 (50.8%)	

Note: PCPs: primary care providers. SD: standard deviation. The significant results were bold.

^1^ The Pearl River Delta includes 9 prefecture-level cities, including Guangzhou, Foshan, Shenzhen, Dongguan, Zhongshan, Zhuhai, Zhaoqing, Jiangmen and Huizhou, and represents an economically developed region. The non-Pearl River Delta includes remaining 12 prefecture-level cities in Guangdong Province, indicating economically underdeveloped region

In Table 2, significant differences in knowledge scores were observed among PCPs with GPs having highest mean score (32.5) and Nurses having lowest mean score (29.0). However, attitudes scores of dementia care did not differ significantly among different occupational groups, with mean scores ranging from 39.6 to 41.0 (*P* = 0.410). Differences in skills scores of dementia care among occupational groups were also not statistically significant (*P* = 0.364). The results of further multiple comparisons are presented in Additional file (Table S1).

**Table 2:** Knowledge, attitudes and skills in dementia care among PCPs and the dementia screening outcomes

Characteristics	General practitioner (*n* = 67, 26.6%)	Public health physician (*n* = 115, 45.6%)	Nurse (*n* = 37, 14.7%)	Social worker (*n* = 33, 13.1%)	*P*
**Dementia care, mean (SD)**					
Knowledge [range 0–50]	32.5 (6.5)	32.2 (6.3)	29.0 (6.3)	29.1 (6.8)	**0.005**
Attitudes [range 10–50]	39.8 (3.9)	40.0 (4.1)	41.0 (3.9)	39.6 (4.1)	0.410
Skills [range 15–75]	52.3 (10.5)	53.0 (9.3)	55.3 (8.3)	51.7 (8.6)	0.364
**Dementia screening outcomes**					
Number of older adults screened, n (%)	801 (28.4%)	1333 (47.2%)	428 (15.2%)	261 (9.2%)	-
Screened positive in first stage, n (%)	163 (20.3%)	316 (23.7%)	104 (24.3%)	71 (27.2%)	0.090
Complete the second stage, n (%)	151 (92.6%)	287 (90.8%)	92 (88.5%)	65 (91.5%)	-
True positive in second stage, n	7	35	17	4	-
PPV, %	4.6	12.2	18.5	6.2	**0.003**

Note: PCPs: primary care providers. SD: standard deviation. True positive: number of people screened positive for dementia and correctly diagnosed as having dementia in the second stage. In the first stage, older people are screened for dementia by PCPs, and those who screen positively go on to the second stage of diagnosis of dementia by a specialist psychiatrist. The significant results were bold.

Percentage of screened positive: $\:\frac{\text{N}\text{u}\text{m}\text{b}\text{e}\text{r}\:\text{o}\text{f}\:\text{p}\text{e}\text{o}\text{p}\text{l}\text{e}\:\text{s}\text{c}\text{r}\text{e}\text{e}\text{n}\text{e}\text{d}\:\text{p}\text{o}\text{s}\text{i}\text{t}\text{i}\text{v}\text{e}\:\text{i}\text{n}\:\text{f}\text{i}\text{r}\text{s}\text{t}\:\text{s}\text{t}\text{a}\text{g}\text{e}}{\text{T}\text{o}\text{t}\text{a}\text{l}\:\text{n}\text{u}\text{m}\text{b}\text{e}\text{r}\:\text{o}\text{f}\:\text{p}\text{e}\text{o}\text{p}\text{l}\text{e}\:\text{s}\text{c}\text{r}\text{e}\text{e}\text{n}\text{e}\text{d}}\times\:100\%$

Positive predictive value (PPV): $\:\frac{\text{N}\text{u}\text{m}\text{b}\text{e}\text{r}\:\text{o}\text{f}\:\text{p}\text{e}\text{o}\text{p}\text{l}\text{e}\:\text{d}\text{i}\text{a}\text{g}\text{n}\text{o}\text{s}\text{e}\text{d}\:\text{p}\text{o}\text{s}\text{i}\text{t}\text{i}\text{v}\text{e}\:\text{i}\text{n}\:\text{s}\text{e}\text{c}\text{o}\text{n}\text{d}\:\text{s}\text{t}\text{a}\text{g}\text{e}\:\left(\text{T}\text{r}\text{u}\text{e}\:\text{p}\text{o}\text{s}\text{i}\text{t}\text{i}\text{v}\text{e}\right)}{\text{N}\text{u}\text{m}\text{b}\text{e}\text{r}\:\text{o}\text{f}\:\text{p}\text{e}\text{o}\text{p}\text{l}\text{e}\:\text{c}\text{o}\text{m}\text{p}\text{l}\text{e}\text{t}\text{e}\text{d}\:\text{t}\text{h}\text{e}\:\text{s}\text{e}\text{c}\text{o}\text{n}\text{d}\:\text{s}\text{t}\text{a}\text{g}\text{e}}\times\:100$%

### The associations of PCPs’ occupations, knowledge, attitudes, and skills in dementia care with dementia screening outcomes

Table 2 also showed the results of dementia screening by different occupations of PCPs. Public health physicians screened the highest percentage of older adults (47.2%), followed by GPs (28.4%). In the first stage, social workers had the highest percentage of older adults screened positive (27.2%), while GPs had the lowest percentage (20.3%), but no significant difference was found (*P* = 0.090). In the second stage, there were significant differences in PPV between different PCPs’ group (*P* = 0.003), with GPs having a PPV of 4.6%, 12.2% for public health physicians, 18.5% for nurses and 6.2% for social workers.

Table 3 showed the associations of PCPs’ occupations, knowledge, attitudes, and skills in dementia care with the positive rate of dementia screening. We did not find a significant association between PCPs’ occupation and positive rate of dementia screening in any of Model 1, 2 or 3. In Model 3, we found the significant associations between PCPs’ attitudes, skills in dementia care and positive rate of dementia screening. The more positive attitudes the PCPs towards dementia care (OR = 0.948, 95%CI: 0.905–0.994), the less likely they were to screen older adults positive for dementia. But the higher the PCPs’ scores on skills in dementia care (OR = 1.024, 95%CI: 1.004–1.046), the more likely they were to screen older adults positive for dementia. Thus, for each unit increase in the PCPs’ score on skill in dementia care, there is a 2.4% increase in the positive rate of dementia screening.

**Table 3:** Associations between PCPs’ occupations and positive rate of screening (*n* = 2823)

Variables	Model 1 ^a^		Model 2 ^b^		Model 3 ^c^	
	OR (95%CI)	*P*	OR (95%CI)	*P*	OR (95%CI)	*P*
**Occupations (Ref. GP)**						
Public health physician	1.204 (0.787, 1.842)	0.391	1.176 (0.768, 1.800)	0.456	1.193 (0.767, 1.854)	0.434
Nurse	1.226 (0.698, 2.154)	0.478	1.194 (0.679, 2.100)	0.538	1.195 (0.618, 2.307)	0.597
Social worker	1.444 (0.786, 2.652)	0.236	1.476 (0.804, 2.713)	0.209	1.321 (0.684, 2.551)	0.408
**Older Adults’ characteristics**						
Age	-	-	**1.050 (1.033**,** 1.068)**	**< 0.001**	**1.051 (1.033**,** 1.069)**	**< 0.001**
Sex (Ref. Female)						
Male	-	-	0.939 (0.765, 1.154)	0.550	0.932 (0.759, 1.145)	0.501
Education level (Ref. Primary school and below)						
Junior school and above	-	-	0.880 (0.701, 1.104)	0.268	0.878 (0.700, 1.103)	0.264
**PCPs’ characteristics**						
Age	-	-	-	-	1.009 (0.980, 1.038)	0.553
Sex (Ref. Female)						
Male	-	-	-	-	0.912 (0.615, 1.352)	0.647
Education level (Ref. Below undergraduate)						
Undergraduage and above	-	-	-	-	1.065 (0.722, 1.571)	0.749
Working years (Ref. < 3 years)						
3–10 years	-	-	-	-	1.001 (0.574, 1.745)	0.998
≥ 10 years	-	-	-	-	0.833 (0.442, 1.570)	0.573
Experienced with dementia care (Ref. No)						
Yes	-	-	-	-	0.801 (0.548, 1.171)	0.252
Working institution (Ref. Rural)						
Urban	-	-	-	-	1.129 (0.741, 1.721)	0.573
Location (Ref. Non-Pearl River Delta)						
Pearl River Delta	-	-	-	-	1.026 (0.687, 1.532)	0.902
**PCPs’ dementia care**						
Knowledge	-	-	-	-	1.006 (0.977, 1.036)	0.698
Attitudes	-	-	-	-	**0.948 (0.905**,** 0.994)**	**0.026**
Skills	-	-	-	-	**1.024 (1.004**,** 1.046)**	**0.021**
**Variance of random effects (PCPs level)**	1.277		1.269		1.174	

Note: Ref. = reference; GP = general practitioner; PCPs = primary care providers; “-”: Not included in the model; OR = odds ratio; CI = confidence interval. The significant results were bold.

^a^ Model 1 only included the variable of PCPs’ occupations.

^b^ Model 2 included the variable of PCPs’ occupations and older adults’ characterstics (age, sex and education level).

^c^ On the basis of Model 2, Model 3 included additional variables of PCPs’ characteristics (age, sex, education level, working years, eperienced with dementia care, working institution and location) and PCPs’ knowledge, attitudes and skills in dementia care

Table 4 showed the associations of PCPs’ occupations, knowledge, attitudes, and skills in dementia care with the PPV. In Model 1, older adults screened positive by public health physicians were 2.927 times (OR = 2.927, 95%CI: 1.091–7.854) more likely to be true positive than GPs, and nurses were 3.712 times (OR = 3.712, 95%CI: 1.141–12.069) more likely than GPs. In Model 2, adjusting for covariates of older adults’ characteristics, only older adults screened positive by nurses (OR = 3.121, 95%CI: 1.044–9.328) were found to be more likely to be truly positive compared to GPs. However, these associations did not persist after further adjusting for PCPs’ characteristics in Model 3. The results of Model 3 showed that the more positive attitudes PCPs towards dementia care (OR = 1.114, 95%CI: 1.007–1.234), the more likely it was that older adults screened positive by PCPs would be truly positive. In further analysing the impact of “having or not having dementia care experience” on knowledge, attitudes and skills of dementia care in the different PCP groups, it was only found in GPs group that GPs with experience in dementia care scored higher on skills than GPs without experience (*P* < 0.001) (see Additional file in Table S2).

**Table 4:** Associations between PCPs’ occupations and positive predict value (*n* = 595)

Variables	Model 1		Model 2		Model 3	
	OR (95%CI)	*P*	OR (95%CI)	*P*	OR (95%CI)	*P*
**Occupations (Ref. GP)**						
Public health physician	**2.927 (1.091**,** 7.854)**	**0.033**	2.393 (0.949, 6.031)	0.064	2.112 (0.785, 5.679)	0.139
Nurse	**3.712 (1.141**,** 12.069)**	**0.029**	**3.121 (1.044**,** 9.328)**	**0.042**	2.995 (0.818, 10.969)	0.098
Social worker	1.382 (0.316, 6.047)	0.668	1.433 (0.357, 5.747)	0.612	1.814 (0.398, 8.277)	0.442
**Older Adults’ characteristics**						
Age	-	-	**1.126 (1.082**,** 1.172)**	**< 0.001**	**1.127 (1.081**,** 1.174)**	**< 0.001**
Sex (Ref. Female)						
Male	-	-	0.788 (0.413, 1.502)	0.469	0.756 (0.391, 1.462)	0.406
Education level (Ref. Primary school and below)						
Junior school and above	-	-	0.590 (0.269, 1.293)	0.187	0.722 (0.321, 1.624)	0.431
**PCPs’ characteristics**						
Age	-	-	-	-	1.011 (0.960, 1.065)	0.674
Sex (Ref. Female)						
Male	-	-	-	-	0.965 (0.425, 2.190)	0.932
Education level (Ref. Below undergraduate)						
Undergraduage and above	-	-	-	-	0.517 (0.237, 1.128)	0.097
Working years (Ref. < 3 years)						
3–10 years	-	-	-	-	2.565 (0.796, 8.263)	0.114
≥ 10 years	-	-	-	-	1.317 (0.358, 4.840)	0.678
Experienced with dementia care (Ref. No)						
Yes	-	-	-	-	**2.649 (1.288**,** 5.445)**	**0.008**
Working institution (Ref. Rural)						
Urban	-	-	-	-	0.669 (0.296, 1.513)	0.335
Location (Ref. Non-Pearl River Delta)						
Pearl River Delta	-	-	-	-	1.451 (0.677, 3.111)	0.338
**PCPs’ dementia care**						
Knowledge	-	-	-	-	1.024 (0.954, 1.099)	0.509
Attitudes	-	-	-	-	**1.114 (1.007**,** 1.234)**	**0.037**
Skills	-	-	-	-	0.990 (0.949, 1.032)	0.626
**Variance of random effects (PCPs level)**	1.448		0.514		0.249	

Note: Ref. = reference; GP = general practitioner; PCPs = primary care providers; “-”: Not included in the model; OR = odds ratio; CI = confidence interval. The significant results were bold.

^a^ Model 1 only included the variable of PCPs’ occupations.

^b^ Model 2 included the variable of PCPs’ occupations and older adults’ characterstics (age, sex and education level).

^c^ On the basis of Model 2, Model 3 included additional variables of PCPs’ characteristics (age, sex, education level, working years, eperienced with dementia care, working institution and location) and PCPs’ knowledge, attitudes and skills in dementia care

## Discussion

Our study systematically investigated the association of dementia screening outcomes of PCPs with their occupations, knowledge, attitudes, and skills in dementia care across 21 prefectures in Guangdong Province. We found that nurses and public health physicians had a higher PPV than GPs, and that positive attitudes and adequate skills were key influencers of PCPs’ performance in dementia screening.

GPs are currently considered to play a major role in identifying potential dementia cases in primary care settings, as they may notice changes in patients’ cognitive performance during routine clinical practice [25]. However, our results indicated no differences in the positive rate of screening among different occupations of PCPs, although all were higher than previous findings in Shenzhen [8]. Furthermore, the PPV for PCPs was less than 20%, with GPs having a particularly low rate of 4.6%, which was significantly lower than that reported in the UK (86%) and more in line with the Indian community (11%) [26, 27]. On the other hand, nurses and public health physicians had a higher PPV than GPs, suggesting that other occupations of PCPs have the potential to improve the accuracy of the screening process. In a community setting, advocating the involvement of different occupations of PCPs in addition to GPs within a referral system may lead to a reduction in false-positive results and improved diagnostic accuracy. Training PCPs of different occupations to detect dementia cases without increasing the existing health workforce may also optimise resource utilisation in resource-limited settings.

Our results also suggested that positive attitudes towards dementia care were an important determinant of PCPs’ performance. In our study, overall knowledge scores for all PCPs tended to be lower than in previous studies [21, 28]. We also found generally low skill scores among PCPs, consistent with a previous study of Beijing GPs [23]. On the other hand, PCPs had higher positive attitude scores compared with previous studies in Beijing and Changsha [23, 28]. Further analysis indicated that only positive attitudes towards dementia were associated with fewer positive outcomes in the first stage and higher PPV. This highlights the importance of positive attitudes among PCPs in reducing false positives in the first stage and achieving accurate screening results. A previous study in European countries have shown that GPs with positive attitudes assume that early diagnosis of dementia is considered to be of value [29]. In China, GPs perceive the identification and diagnosis of dementia as the responsibility of specialists [15]. Furthermore, Chinese GPs may be hesitant or unable to detect dementia, relying on neurophysiological questionnaires and a lack of objective measures [30]. In this study without adjusting for attitudes, a factor that has a significant effect on PPV, we found that nurses and public health physicians had higher PPV that GPs. We speculate that this may be because the organisation of the screening programme is part of the public health system, and that nurses and public health physicians have a higher level of attention in dementia screening and show a more positive attitude. These findings highlight the need for efforts to promote positive attitudes towards dementia care among PCPs.

Our findings indicate that experience in dementia care may partially explain the differences in PPV among PCPs. This implies that experience plays a role in improving PCPs’ ability to accurately identify true positive cases, potentially leading to higher PPV. Further analysis suggested that GPs with experience in dementia care achieved higher skill scores, highlighting the importance of providing opportunities for PCPs to gain practical experience in dementia care. However, knowledge, attitudes, and skills scores did not significantly differ between public health physicians, nurses, and social workers with and without experience in dementia care. For nurses and social workers, limited exposure to dementia cases may limit the impact of experience in dementia care. For public health physicians, while their role in mental health prevention and treatment is officially accredited, China has not yet established standardized clinical training system for them [31]. Consequently, despite also having more exposure to dementia cases, they may possess less direct clinical experience than GPs, which explains their comparable skill scores. Moreover, we found that better dementia care skills were associated with a higher positive rate of screening in the first stage, and to some extent this could reduce the number of false-negative cases failing to progress to the second stage of diagnosis. Thus, investment in continuing education and professional skills training can improve the effectiveness of dementia screening and management, particularly for professionals with limited experience.

Rates of dementia vary across geographic differences like levels of economic development and urban/rural areas [32]. In this study, there were significant differences in the workplace (urban or rural) and location (PRD or NPRD) of the PCPs responsible for dementia screening, which may have an impact on our research objectives. Therefore, by adjusting for these geographic factors, we were able to get more accurate insights into our principal focus. Another strength of the study is that we matched PCPs and screened older adults, and adjusted for their characteristics simultaneously, ensuring a more accurate assessment of associations. However, there were some limitations to this study. We used self-rated scale questionnaires to assess the level of knowledge, attitudes and skills of PCPs, which may not fully capture the true level due to social expectations and subjective interpretation of skill levels. In addition, the questionnaire on skills of dementia care was developed for GPs and may be biased on other PCPs. Future studies are needed to include objective assessments by relevant people, such as colleagues of PCPs. Second, although the diagnosis in the second stage was made by qualified psychiatrists, the results may still be susceptible to subjectivity. To obtain more accurate measures, future research should carry out further clinical verification or standardised protocols to improve them. In addition, this study only focused on Guangdong, which limited the representativeness of Chinese PCPs. Future studies should consider including PCPs from a wider range of regions within China.

## Conclusion

Our study focused on assessing the screening performance of PCPs according to their occupations. We found that nurses and public health physicians demonstrated adequate performance with high PPV. Importantly, our study identified that positive attitudes and adequate skills towards dementia care are the key factors influencing their performance. PCPs with experience in dementia care showed improved performance, particularly GPs with experience who had higher skill scores. In community settings, systematically promoting dementia-care skills training and positive attitudes towards dementia care is important to improve the effectiveness of dementia screening and management. In addition, involving PCPs from occupations other than GPs in participating in dementia screening may not only improve the effectiveness of dementia screening but also optimize resource utilization in resource-limited settings.

## Electronic supplementary material

Below is the link to the electronic supplementary material.

Supplementary Material 1

## Data Availability

The datasets used and/or analysed during the current study are available from the corresponding author on reasonable request.

## Abbreviations

AD8: Ascertain Dementia 8
CSI-D: Community Screening Interview for Dementia
DCAS: Dementia Care Assessment Scale
DKAS: Dementia Knowledge Assessment Scale
GPs: General practitioners
NPRD: Non-Pearl River Delta
PCP: Primary care provider
PPV: Positive predictive value
PRD: Pearl River Delta
