# Supplementary material for: Variation in dementia screening outcomes: the influence of primary care providers’ occupations and knowledge, attitudes, skills
Source: BMC Prim Care. 2025 May 28;26:187. doi: 10.1186/s12875-025-02886-y (PMC12117673; doi:10.1186/s12875-025-02886-y)
Supplement: Supplementary file 1 — Supplementary Material 1 [file 12875_2025_2886_MOESM1_ESM.docx]

**Table S1. Comparison of knowledge, attitudes, and skills scores by PCPs’ occupations**

| **Comparison group** | **Knowledge Score** | **Attitudes Score** | **Skills Score** |
| --- | --- | --- | --- |
| **General practitioner vs. Public health physician** | | | |
| *t* | 0.291 | -0.371 | -0.447 |
| adjusted *P* ^1^ | 0.999 | 0.999 | 0.999 |
| **General practitioner vs. Nurse** | | | |
| *t* | 2.687 | -1.556 | -1.598 |
| adjusted *P* ^1^ | 0.053 | 0.744 | 0.684 |
| **General practitioner vs. Social worker** | | | |
| *t* | 2.407 | 0.214 | 1.782 |
| adjusted *P* ^1^ | 0.115 | 0.999 | 0.476 |
| **Public health physician vs. Nurse** | | | |
| *t* | 2.697 | -1.365 | -1.418 |
| adjusted *P* ^1^ | 0.054 | 0.999 | 0.966 |
| **Public health physician vs. Social worker** | | | |
| *t* | 2.375 | 0.506 | 0.757 |
| adjusted *P* ^1^ | 0.129 | 0.999 | 0.999 |
| **Nurse vs. Social worker** | | | |
| *t* | -0.056 | 1.481 | 1.782 |
| adjusted *P* ^1^ | 0.999 | 0.858 | 0.476 |

Note: PCPs: Primary care providers. ^1^ Bonferroni method was used to adjust the *P* value.

**Table S2. Comparison of knowledge, attitudes and skills scores by PCPs’ occupations with and without experience in dementia care**

| Whether experienced with dementia care | N | Knowledge Score  Mean (SD) | Attitudes Score  Mean (SD) | Skills Score  Mean (SD) |
| --- | --- | --- | --- | --- |
| General practitioner | | | | |
| Yes | 26 | 34.3 (6.5) | 40.3 (3.3) | **57.8 (8.1)** |
| No | 41 | 31.3 (6.2) | 39.5 (4.3) | **48.8 (10.4)** |
| *P* |  | 0.064 | 0.429 | **< 0.001** |
| Public health physician | | | | |
| Yes | 48 | 32.1 (6.8) | 40.0 (3.6) | 54.4 (8.8) |
| No | 67 | 32.3 (6.0) | 40.1 (4.4) | 52.0 (9.6) |
| *P* |  | 0.854 | 0.859 | 0.181 |
| Nurse | | | | |
| Yes | 12 | 27.3 (6.0) | 40.7 (3.7) | 58.0 (9.5) |
| No | 25 | 29.8 (6.4) | 41.2 (4.0) | 54.0 (7.5) |
| *P* |  | 0.256 | 0.700 | 0.169 |
| Social worker | | | | |
| Yes | 5 | 28.6 (7.7) | 39.8 (4.5) | 54.4 (5.4) |
| No | 28 | 29.1 (6.8) | 39.6 (4.1) | 51.2 (9.0) |
| *P* |  | 0.872 | 0.911 | 0.447 |

Note: PCPs: Primary care providers; SD: Standard deviation. The significant results were bold.

STROBE Statement—Checklist of items that should be included in reports of ***cross-sectional studies***

|  | **Item No** | **Recommendation** | **Page No** |
| --- | --- | --- | --- |
| **Title and abstract** | 1 | (*a*) Indicate the study’s design with a commonly used term in the title or the abstract | ☑Page 1 |
|  |  | (*b*) Provide in the abstract an informative and balanced summary of what was done and what was found | ☑Page 2-3 |
| **Introduction** | | | |
| Background/rationale | 2 | Explain the scientific background and rationale for the investigation being reported | ☑Page 4-6 |
| Objectives | 3 | State specific objectives, including any prespecified hypotheses | ☑Page 5, Line 115-117 |
| **Methods** | | | |
| Study design | 4 | Present key elements of study design early in the paper | ☑Page 6 |
| Setting | 5 | Describe the setting, locations, and relevant dates, including periods of recruitment, exposure, follow-up, and data collection | ☑Page 6-7 |
| Participants | 6 | (*a*) Give the eligibility criteria, and the sources and methods of selection of participants | ☑Page 7-8 |
| Variables | 7 | Clearly define all outcomes, exposures, predictors, potential confounders, and effect modifiers. Give diagnostic criteria, if applicable | ☑Page 8-10 |
| Data sources/ measurement | 8* | For each variable of interest, give sources of data and details of methods of assessment (measurement). Describe comparability of assessment methods if there is more than one group | ☑Page 7-9 |
| Bias | 9 | Describe any efforts to address potential sources of bias | Not Discuss |
| Study size | 10 | Explain how the study size was arrived at | ☑Page 7 |
| Quantitative variables | 11 | Explain how quantitative variables were handled in the analyses. If applicable, describe which groupings were chosen and why | ☑Page 10 |
| Statistical methods | 12 | (*a*) Describe all statistical methods, including those used to control for confounding | ☑Page 10-11 |
|  |  | (*b*) Describe any methods used to examine subgroups and interactions | Not Discuss |
|  |  | (*c*) Explain how missing data were addressed | Not Discuss |
|  |  | (*d*) If applicable, describe analytical methods taking account of sampling strategy | Not Discuss |
|  |  | (*e*) Describe any sensitivity analyses | Not Discuss |
| **Results** | | | |
| Participants | 13* | (a) Report numbers of individuals at each stage of study—e.g. numbers potentially eligible, examined for eligibility, confirmed eligible, included in the study, completing follow-up, and analysed | ☑Page 7-8 |
|  |  | (b) Give reasons for non-participation at each stage | ☑Page 7-8 |
|  |  | (c) Consider use of a flow diagram | ☑Page 27 |
| Descriptive data | 14* | (a) Give characteristics of study participants (eg demographic, clinical, social) and information on exposures and potential confounders | ☑Page 11-12 |
|  |  | (b) Indicate number of participants with missing data for each variable of interest | Not Discuss |
| Outcome data | 15* | Report numbers of outcome events or summary measures | ☑Page 12-14 |
| Main results | 16 | (*a*) Give unadjusted estimates and, if applicable, confounder-adjusted estimates and their precision (e.g. 95% confidence interval). Make clear which confounders were adjusted for and why they were included | ☑Page 12-14 |
|  |  | (*b*) Report category boundaries when continuous variables were categorized | Not Discuss |
|  |  | (*c*) If relevant, consider translating estimates of relative risk into absolute risk for a meaningful time period | Not Discuss |
| Other analyses | 17 | Report other analyses done—eg analyses of subgroups and interactions, and sensitivity analyses | ☑Page 13-14 |
| **Discussion** | | | |
| Key results | 18 | Summarise key results with reference to study objectives | ☑Page 14 |
| Limitations | 19 | Discuss limitations of the study, taking into account sources of potential bias or imprecision. Discuss both direction and magnitude of any potential bias | ☑Page 16-17 |
| Interpretation | 20 | Give a cautious overall interpretation of results considering objectives, limitations, multiplicity of analyses, results from similar studies, and other relevant evidence | ☑Page 14-17 |
| Generalisability | 21 | Discuss the generalisability (external validity) of the study results | ☑Page 17 |
| **Other information** | | | |
| Funding | 22 | Give the source of funding and the role of the funders for the present study and, if applicable, for the original study on which the present article is based | ☑Page 18-19 |

*Give information separately for exposed and unexposed groups.
